# Dev-ResNet: automated developmental event detection using deep learning

**DOI:** 10.1242/jeb.247046

**Published:** 2024-05-29

**Authors:** Ziad Ibbini, Manuela Truebano, John I. Spicer, Jamie C. S. McCoy, Oliver Tills

**Affiliations:** Marine Biology and Ecology Research Centre, School of Biological and Marine Sciences, University of Plymouth, Drake Circus, Plymouth PL4 8AA, UK

**Keywords:** Computer vision, Video classification, Convolutional neural networks, Bioimage analysis, Heterochrony

## Abstract

Delineating developmental events is central to experimental research using early life stages, permitting widespread identification of changes in event timing between species and environments. Yet, identifying developmental events is incredibly challenging, limiting the scale, reproducibility and throughput of using early life stages in experimental biology. We introduce Dev-ResNet, a small and efficient 3D convolutional neural network capable of detecting developmental events characterised by both spatial and temporal features, such as the onset of cardiac function and radula activity. We demonstrate the efficacy of Dev-ResNet using 10 diverse functional events throughout the embryonic development of the great pond snail, *Lymnaea stagnalis*. Dev-ResNet was highly effective in detecting the onset of all events, including the identification of thermally induced decoupling of event timings. Dev-ResNet has broad applicability given the ubiquity of bioimaging in developmental biology, and the transferability of deep learning, and so we provide comprehensive scripts and documentation for applying Dev-ResNet to different biological systems.

## Teaser

**Summary:** Dev-ResNet is a small and efficient 3D convolutional neural network capable of automated detection of developmental events with both spatial and temporal components.

## INTRODUCTION

Embryonic development is a fundamental period of life and plays a major role in the evolution of novel phenotypes. Throughout its long history as a focus for biological research (Darwin, 1859; Haeckel, 1866; Garstang, 1922; Gould, 1977), effectively quantifying the continuous process of dynamic temporal and spatial change at the phenotypic level has been a recurring challenge for the field. For over a century, biologists routinely have used developmental event timings as their ‘time-stamps’. Events range from the onset of growth of morphological features, through to physiological systems such as the onset of function in cardio-respiratory systems. They have been broadly used in experimental research, including areas such as ecology, ecotoxicology and, most prominently, evolutionary biology. In the last of these, developmental events are used to compare the development of different species, as surrogates for chronological and developmental time, and to identify heterochronies, changes in the timing of developmental events between an ancestor and their descendant (Gould, 1977; Spicer and Rundle, 2006). Heterochronies are a frequent occurrence in living things and posited as key drivers of evolutionary change (MacKinney and MacNamara, 1991; McNamara and McKinney, 2005; Spicer et al., 2011). Developmental event timings have also proven invaluable in the comparison of environmental sensitivity between stressors, stages of development and species (Burggren, 2021; Rundle and Spicer, 2016).

The use and delineation of developmental event timings has attracted controversy (Richardson and Keuck, 2022), but they remain widely used with few viable alternatives. A key strength of developmental event timings is the ability to compare markedly different aspects of development across the animal kingdom, and across a range of environmental conditions. Much of the controversy centres on the precise definition of the characteristics of individual events. No two embryos are identical (Spicer and Rundle, 2006), and therefore neither are the appearances of their developmental events. Furthermore, identifying developmental events can be time consuming and highly subjective, with consequences for the accuracy and reproducibility of the metrics used (Love, 2010). However, advances in methods for analysis of bioimaging datasets are presenting new opportunities. The use of deep learning for bioimage analysis has increased significantly, owing to its potential for generating models capable of solving complex image analysis tasks that are increasingly at the limits of human perception (Nogare et al., 2023).

Deep learning methods typically used for developmental studies involving classification of different embryonic phenotypes involve the use of convolutional neural networks (CNNs) (Hallou et al., 2021). CNNs have been applied to the identification of developmental stages (Liu et al., 2019; Pond et al., 2021), embryonic selection and health measurement for *in vitro* fertilisation (Chen et al., 2019; Khosravi et al., 2019; Louis et al., 2021), the detection of deformations in embryonic phenotypes (Ishaq et al., 2017; Čapek et al., 2023), and the prediction of developmental time (Jones et al., 2023; Toulany et al., 2023). However, many of these deep learning methods rely on models trained on 2D images (e.g. Liu et al., 2019; Khosravi et al., 2019; Ishaq et al., 2017; Pond et al., 2021; Čapek et al., 2023), and therefore rely solely on morphological features to distinguish between different embryonic phenotypes of interest. Identifying developmental events in embryos, particularly those that are physiological, such as movement and cardiac function, require spatio-temporal data, i.e. videos or sequences of images. Yet, the potential for a model capable of integrating both spatial and temporal information, to perform automated and accurate detection of a diverse range of developmental events throughout development, remains untested.

Here, we introduce Dev-ResNet (https://github.com/EmbryoPhenomics/dev-resnet), a 3D convolutional neural network model capable of detecting both morphological and physiological developmental events throughout embryonic development, drawing on 3D spatio-temporal information. Dev-ResNet was validated using the highly spatially and functionally dynamic embryonic development of the great pond snail, *Lymnaea stagnalis* (Kuroda and Abe, 2020)*.* Training of Dev-ResNet was performed on manually labelled videos of 67 embryos (comprising 23,283 videos), to predict the occurrence of 10 different developmental events, ranging from gastrulation to the onset of heart function and crawling. We also tested the applicability of Dev-ResNet to experimental contexts involving different environmental conditions, by imaging the entire development of 405 embryos (comprising 154,082 videos) across a wide range of chronic thermal assays, demonstrating both considerable thermal plasticity in the timing of developmental events and consistent efficacy of the model.

## MATERIALS AND METHODS

### Model design

**Fig. 1.**
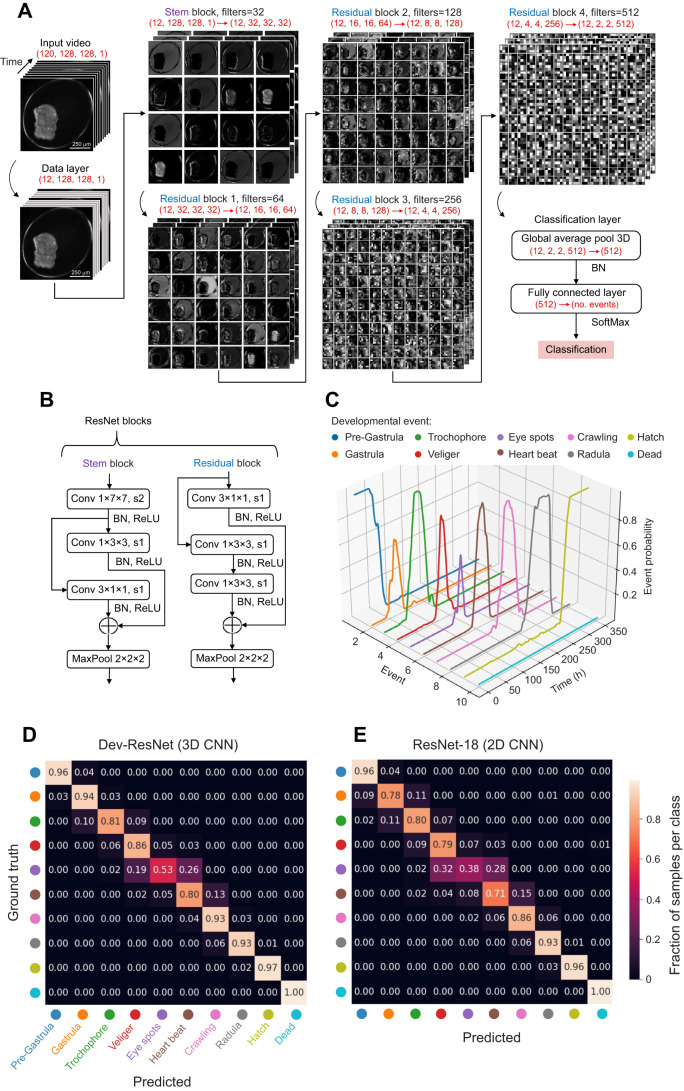
**Dev-ResNet: a 3D ResNet network architecture for detecting the onset of developmental events.** (A) Feature maps at each stage of the network are visualised, and these are based on the sample video shown at the start (see ‘Input video’). The number of filters at each ResNet layer corresponds to the number of convolutional filters associated with that layer. Red text beneath each of the sub-headers denotes the dimensions at a given layer and any changes in resolution achieved by a given layer. (B) Layer diagrams for the stem and residual blocks described in A, where kernel sizes associated with 3D Conv and MaxPool layers are described by the length×width×height notation (e.g. Conv 1×3×3), and stride sizes associated with 3D Conv layers are described by s(*x*) notation (e.g. s1 or s2). Residual connections in the 3D ResNet layers are shown using the addition symbol at the end of each block. (C) An example developmental event sequence for a single embryo, as predicted by Dev-ResNet, is shown for *L. stagnalis* development at 20°C, where event probabilities (*z*-axis) correspond to the confidence of the model in a given event classification. (D,E) Confusion matrices comparing counts between ground truth and predicted developmental event labels on the testing dataset for Dev-ResNet (D) and an equivalent 2D convolutional neural network (CNN), ResNet-18 (E), trained on the training dataset with selective data augmentation, i.e. class imbalances were removed by applying augmentation to varying degrees depending on the representation of a given class in the original data. The following are the number of sample videos associated with each event in the testing data: Pre-Gastrula *n*=280, Gastrula *n*=255, Trochophore *n*=206, Veliger *n*=232, Eye spots *n*=47, Heart beat *n*=127, Crawling *n*=333, Radula *n*=475, Hatch (*n*=257) and Dead *n*=126. The axis tick colours assigned to each event are described in D. BN, batch normalisation; ReLU, rectified linear unit; MaxPool, 3D maximum pooling layer; FCN, fully connected network; Conv, 3D convolutional layer.

A key point of difference in Dev-ResNet is that instead of using 2D images as inputs and relying solely on morphological differences for delineating discrete developmental events, Dev-ResNet uses videos of developing embryos converted to a 3D stack of images for input, thus enabling both spatial and temporal features to be used for the detection of developmental events (Fig. 1A).

We opted to use an input video resolution of length=120, width=128, height=128, channels=1, and to perform a large temporal stride (*n*=10) to downsample video to a resolution of length=12, width=128, height=128, channel=1, i.e. the model only processes 1 out of every 10 video frames, thus increasing computational efficiency whilst retaining global temporal features (Fig. 1A). At a typical recording frame rate of 30 frames s^−1^, this temporal stride means that the model makes use of three video frames per second of footage.

The backbone of Dev-ResNet is composed of a 3D ResNet architecture (Fig. 1A), whereby 2D spatial convolutions are coupled with 1D temporal convolutions to produce 3D features at each residual block in the model (Fig. 1A,B). This approach to making 3D residual blocks is often termed Conv2plus1D (Tran et al., 2018), and has been shown to achieve both increased computational efficiency and comparable, or greater, accuracy when compared with using pure 3D convolutional blocks (Tran et al., 2018).

Finally, a 3D global pooling layer and a fully connected layer is used to perform classification (Fig. 1A) – a classification block that is commonly used in many state-of-the-art 3D classification models (e.g. SlowFast, Feichtenhofer et al., 2019; MoViNet, Kondratyuk et al., 2021).

### Embryo collection

Adult *Lymnaea stagnalis* (Linnaeus 1758) (*n*=25) were sampled from a laboratory stock population and transferred to individual aquaria (*n*=25, 1.5 l, 20°C) filled with aerated artificial pond water (APW; chemical composition: CaSO_4_ 120 mg l^−1^, MgSO_4_ 245 mg l^−1^, NaHCO_3_ 192 mg l^−1^, KCl 8 mg l^−1^) with a 12 h:12 h light:dark regime. The stock population at University of Plymouth was founded from a culture of the RENILYS strain (donated from IRNA, Rennes, France) established in October 2013 and maintained for multiple generations in large aquaria (*n*=3, 25 l, 18–22°C, APW). Individuals of the culture used for the present study were fed lettuce *ad libitum* and water changes were carried out 1–2 times per week. Egg masses were removed from aquaria using double layer laminate plastic within 24 h of deposition, and inspected under low power magnification (×10–40, HM-4, Microtech); egg masses containing embryos that had not developed beyond the 2nd cell division were extricated from the egg mass and transferred to 96-well microtitre plates (Nunc, Microwell, 350 µl per well) for bioimaging.

### Thermal assays and bioimaging

*Lymnaea stagnalis* embryos (*n*=405) were exposed to chronic thermal assays at 15°C (*n*=58), 17.5°C (*n*=60), 20°C (*n*=38), 22.5°C (*n*=55), 25°C (*n*=38), 27.5°C (*n*=59), 30°C (*n*=37) and 32.5°C (*n*=60), and imaged for 20 s (1024×1026 pixels, 30 frames s^−1^, 8-bit depth, ×200 magnification) every hour from first cell division until hatching. This was achieved using the *OpenVIM* system, an open-source software-controlled video-microscope with a robotic *X* and *Y* motorized stage for high-throughput time-lapse imaging of developing embryos (Tills et al., 2018). Embryos were held in 96-well microtitre plates housed within jacket incubation chambers maintained at the treatment-specific constant temperature, located within OpenVIM. Water levels within microtitre plates were checked 1–2 times per day during imaging and topped up with Milli-Q (Merck, Germany) water as required. The OpenVIM system uses *MicroManager* (Edelstein et al., 2010) to perform image acquisitions, acquiring videos as a sequence of TIFF files. We developed a Python script for concatenating these TIFF files into single time-lapse videos for each individual embryo (see https://github.com/EmbryoPhenomics/fast_img_compile/). This facilitated more efficient creation of the training dataset used for training Dev-ResNet. Finally, a pre-trained *Xception* (Chollet, 2017) model was used for egg localisation to limit video footage to a 2D bounding box of just the egg. For more information, please see https://github.com/EmbryoPhenomics/egg_detection/.

### Training dataset

The video dataset captured above was subsampled to a smaller dataset for manual annotation (*n*=67 individual embryos), with 4−18 replicates per chronic temperature treatment. The hour at which each developmental event occurred was manually recorded by viewing the time-lapse video files for each embryo (see Fig. S1 and Table S1 for event images and descriptions, respectively). These ground-truth event timings were used to create multi-class annotations, whereby a given video would be labelled as the previous event up until the subsequent event occurred. Finally, we subsampled each time point video to every 10th frame of the first 120 video frames (i.e. 4 s of video) and subsequently converted these subsampled videos to GIF files.

The final training dataset size before data augmentation was *n*=23,283 videos, where each video had resolution length=12, width=128, height=128, channels=1. This dataset was split further into training, validation and testing datasets using an 80:10:10 split, i.e. 80% samples for training and 10% samples each for validation and testing.

The *ImgAug* Python package (https://github.com/aleju/imgaug) was used to apply data augmentation to the training video dataset, where augmentations were deterministic on a sample-specific basis, i.e. the same set of augmentations were applied to each frame in a video sample. Data augmentations applied to the training dataset were: horizontal and vertical flips, amplification (2×) or reduction (0.5×) in pixel values, as well as salt and pepper noise, and Gaussian blur. These data augmentations were used to upsample the dataset size and remove class imbalances between the number of videos associated with a given developmental event. We upsampled all classes to the same number of samples, and the following are the number of augmented videos for classes that were upsampled: pre-gastrula *n*=846, gastrula *n*=1042, trochophore *n*=1170, veliger *n*=1310, eye spots *n*=2652, heart beat *n*=1960, crawling *n*=405, hatch *n*=899 and dead *n*=1835. This type of selective data augmentation facilitated training a far more performant model than non-selective data augmentation (i.e. number of training samples increases but class imbalances remain) (Fig. S2A–C). The full video dataset, including annotations, is available from Zenodo (https://zenodo.org/record/8214975).

### Training protocol

Training was performed with sparse categorical cross-entropy loss and Adam optimisation with a fixed learning rate of 1e−6. We arrived at this learning rate through testing of other learning rates as well as adaptive learning rate schedulers. We found that a fixed learning rate of 1e−6 provided the most stable training process as well as the highest testing accuracy. Models were trained for 50 epochs with a batch size of 32 videos.

### Identification of developmental event timings

We identified developmental event timings from probability trajectories associated with each event. Probability trajectories (e.g. Fig. 1C) are derived from applying Dev-ResNet to each time point video in a given embryo's development, which thus produces time-series classifications for each developmental event. We then used two different approaches for identifying developmental event timings from event probability trajectories generated by the final classification layer of Dev-ResNet (Fig. 1C). Where event probability trajectories resulted in a peak corresponding to the onset of that event (Gastrula through to Crawling in Fig. 1C), we simply used the time at the maximum probability (i.e. the peak) as the time of onset. Conversely, where an event probability trajectory rapidly increased to a plateau (Hatch and Dead in Fig. 1C), a threshold was also applied but, instead, the first hour at which Dev-ResNet assigned a probability higher than the threshold was then used as the time of onset. For this latter approach, the following probability thresholds were used: Radula 0.6, Hatch 0.4 and Dead 0.3. Differences in threshold values are due to differences in the upper limits of event probabilities produced by Dev-ResNet for each event (see Fig. 1C). These thresholds were used across all treatments and were identified by comparing predicted event times versus ground truth data for different threshold values. Thus, we recommend that users of Dev-ResNet perform manual annotations on a sample of their dataset to identify ground-truth timings before computing threshold values.

### Comparison against equivalent 2D architecture

We performed benchmarking comparisons between our 3D ResNet architecture and an equivalent 2D architecture (ResNet-18; He et al., 2016) to establish the importance of temporal information in the detection of developmental events. We trained and tested the ResNet-18 architecture using the same training parameters outlined above for the 3D model (see ‘Training protocol’), but using only the first frame from each video sample in the training and testing dataset created for the 3D Dev-ResNet model. In doing so, the 2D ResNet model saw the exact same examples during training and testing as the 3D ResNet model, thus enabling us to exclusively test the importance of temporal information in this classification task. Training and testing of both models (2D and 3D) was carried out 3 times with different random seeds (see https://github.com/EmbryoPhenomics/dev-resnet/blob/v0.2/train_2d.ipynb), and an average was computed across all iterations for comparisons of top-1 classification accuracy (proportion of classifications where the predicted label exactly matches the ground truth label) and confusion matrices.

### Comparison between different temporal strides and video lengths

To test for the effect of temporal stride and video length on final classification accuracy, we re-trained Dev-ResNet on videos of a range of temporal strides (3, 5 and 10 frame stride, but with the same 4 s length; see https://zenodo.org/records/10702658) and time point video lengths (4, 8 and 16 s, but with the same 10 frame stride; see https://zenodo.org/records/10719261). The training, validation and testing datasets for each combination of parameters (stride and length) were sourced from the same dataset outlined above, only the number of frames associated with each video sample was different. Selective augmentation was used on all training datasets. We used the exact same training parameters outlined above for the original model (see https://github.com/EmbryoPhenomics/dev-resnet/blob/v0.2/train_2d.ipynb), and performed training across three different random seeds for the comparison of top-1 classification accuracy and confusion matrices (Fig. S3A–E). Statistical comparisons of top-1 classification accuracy were performed separately for temporal stride and video length using a one-way ANOVA in R v4.3.2.

### Visualisation of output neurons as 2D embeddings

Leveraging CNNs’ ability to distil relevant features from a multi-dimensional input to a single-dimensional output, we re-trained Dev-ResNet using triplet semi-hard loss (Schroff et al., 2015) to investigate the relative differences, or similarities, between embryos at different developmental events. Triplet loss trains a model to minimise Euclidean distances between samples of the same class, and maximise distances between samples of different classes. Here, we replaced the final classification layer of Dev-ResNet with a linear, fully connected layer with *L*_2_ normalization. This fully connected layer had the same number of neurons as the preceding average pooling layer. We subsequently trained this modified model with Adam optimisation with a learning rate of 1e−3 for 20 epochs. We then applied this model to the testing dataset and processed the output using both principal component analysis (PCA) and the UMAP algorithm (McInnes et al., 2018) with default parameters, to project the model output to a 2D space for visualisation purposes (see https://github.com/EmbryoPhenomics/dev-resnet/blob/v0.2/train_2d.ipynb). The following are the key default parameters used for UMAP: *n*_neighbours_=15, *n*_components_=2, metric=euclidean, min_dist=0.1, though the full list of parameters can be found at https://github.com/lmcinnes/umap/blob/master/umap/umap_.py#L1410.

## RESULTS AND DISCUSSION

### Accurate detection of development events with Dev-ResNet

Manual detection of physiological or behavioural developmental events (e.g. onset of cardiac activity or crawling) typically requires real-time spatio-temporal information for confident assessments. Despite this, still images are typically used to train 2D CNNs for classification tasks in developmental studies (Pond et al., 2020; Chen et al., 2019; Čapek et al., 2023; Ishaq et al., 2017; Louis et al., 2021).

To test the importance of temporal information for detecting developmental events, we compared results with a 2D equivalent (ResNet-18; He et al., 2016) of Dev-ResNet. Dev-ResNet achieved significantly higher classification accuracy than the 2D equivalent (90.3% versus 87.1%) (*t*_4_=−8.74, *P*<0.001, for top-1 accuracy). Comparison of confusion matrices (Fig. 1D,E) further emphasised the value of a 3D model approach for physiological or behavioural events such as the onset of cardiac activity (ResNet-18: 71%, Dev-ResNet: 80%; Fig. 1D,E) and crawling (ResNet-18: 86%, Dev-ResNet: 93%; Fig. 1D,E). Whilst morphological characteristics undoubtedly contribute to event classifications, particularly because *L. stagnalis* embryos grow considerably during development, we show that incorporating temporal information via Dev-ResNet significantly enhances classification accuracy. However, adjustments to temporal stride (*F*_2,6_=3.175, *P*=0.115) and video length (*F*_2,6_=2.712, *P*=0.145) did not significantly impact classification accuracy of Dev-ResNet (Fig. S3A–E).

### Visualising the continuous process of development

**Fig. 2.**
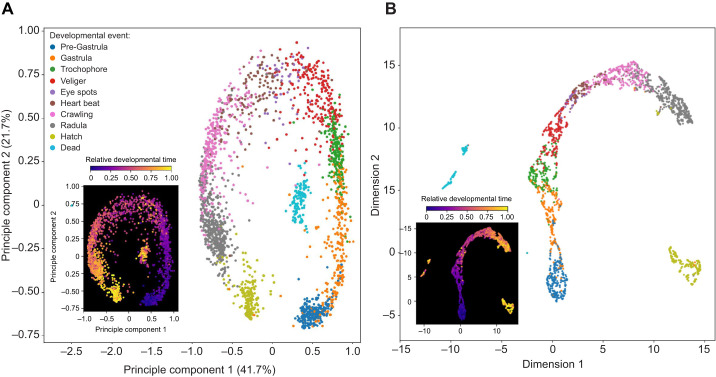
**2D embeddings derived from the separate application of principal component analysis (PCA) and UMAP to the output of Dev-ResNet trained using triplet semi-hard loss.** Each point in the PCA (A) and UMAP (B) 2D embeddings represents a single video of a single embryo from the testing dataset (*n*=2339). The insets showcase the relative developmental time associated with each data point, normalised on the basis of time of hatch or death.

Developmental events mark specific points in a process that is otherwise a continuum of spatial and functional change. However, effectively visualising differences between developmental periods associated with key events remains challenging. Here, visualization of the single dimensional output from Dev-ResNet trained with Triplet loss revealed significant clustering between developmental events, but also a continuum aligned with developmental time (Fig. 2).

Videos associated with different developmental events exhibited overlap when visualized using both linear (PCA) (Fig. 2A) and non-linear (UMAP) (Fig. 2B) dimensionality reductions but mainly for consecutive developmental events, forming a continuum from early- to late-stage embryos with clustering within each event (Fig. 2). This developmental continuum is further reinforced when points in these 2D embeddings are coloured by relative time of occurrence, revealing a continuous gradation of colour (Fig. 2). Distinct clusters in this continuum are of videos containing hatched (Fig. 2B) or dead embryos (Fig. 2A,B), which are probably driven by the marked visual differences associated with these events, given that videos of hatchlings contain an empty egg capsule and dead embryos are rapidly colonised by microbes. These data underscore a crucial consideration in the study of developmental events: physiological and morphological change through development is continuous, rather than discrete (Fig. 2). Deep learning has enabled the characterisation of the continuous nature of development in other species, such as zebrafish (e.g. Toulany et al., 2023) and thus Dev-ResNet could provide a new objective tool with which to characterise both morphological and physiological development.

### Dev-ResNet can detect developmental period-specific thermal sensitivities

**Fig. 3.**
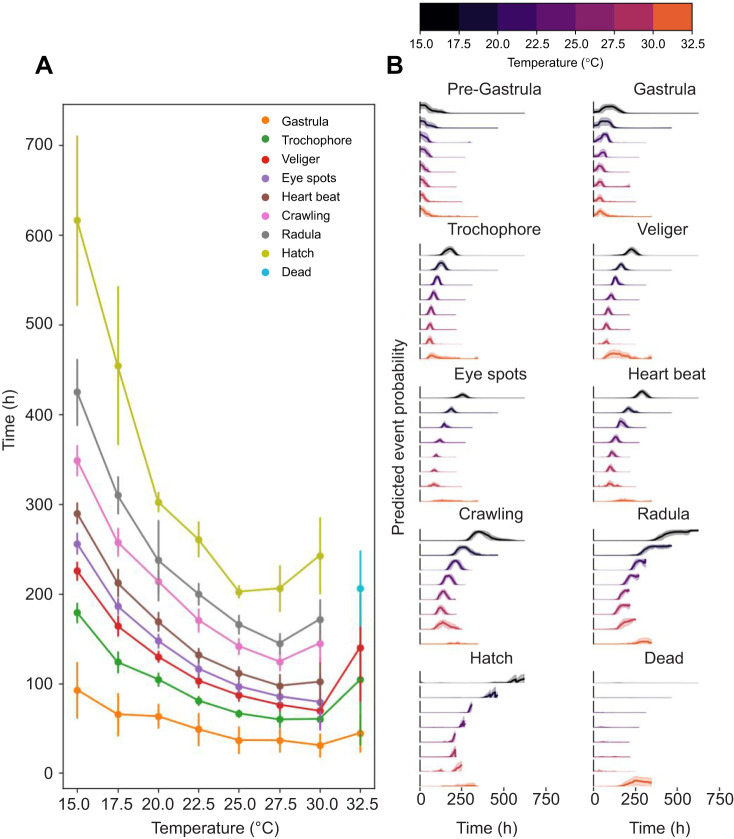
**Timing of developmental events and time-series event probabilities of *Lymnaea stagnalis* embryos incubated at a range of chronic temperatures.** (A) Timing of developmental events (means±s.d.) at: 15°C (*n*=58), 17.5°C (*n*=60), 20°C (*n*=38), 22.5°C (*n*=55), 25°C (*n*=38), 27.5°C (*n*=59), 30°C (*n*=37) and 32.5°C (*n*=60). (B) Time-series event probabilities at the indicated temperatures. All *y*-axes have limits of min=0 and max=1. Solid lines represent the mean whilst shaded regions represent ±1 s.d. Note that Pre-Gastrula classifications were excluded in mean event timings as we use this classification for samples associated with development prior to gastrulation, rather than as an event itself.

To test whether a deep learning approach can detect treatment-level effects in developmental event timing, we compared the entire development of *L. stagnalis* embryos (*n*=405) incubated to a range of chronic temperatures. Dev-ResNet successfully automated the analysis of this video dataset and detected developmental period-specific thermal sensitivities: increasing temperature accelerated developmental rate to all developmental events (Fig. 3), but comparison between early and late event timings indicated reductions in the optimum temperature (*T*_opt_, temperature at which developmental rate is highest) (Fig. 3A). Such reductions in *T*_opt_ for developmental rate highlight heightened thermal sensitivity to upper extreme temperatures in later developmental periods, potentially as a result of increased costs associated with continued heat shock responses throughout development under chronically elevated temperatures (Tomanek, 2010).

### Potential use and limitations

Organismal development, with its inherent physiological diversity and multifaceted time-dependent characteristics has long posed a challenge to researchers who required simplistic approaches to its study. 3D CNNs integrate spatial and temporal features, making them highly relevant to a range of biological characteristics, including morphology and physiology. We found that a single 3D CNN, Dev-ResNet, accurately identified developmental events for which the features of interest are highly different in their appearance and temporal characteristics. Furthermore, Dev-ResNet effectively measured these developmental event timings across a broad range of chronic temperatures, providing a granular resolution in embryonic thermal sensitivity. A key differentiator between Dev-ResNet and other CNNs models for developmental studies (e.g. Jones et al., 2023; Toulany et al., 2023) is the use of discrete functional developmental events, which can change in timing independently of one another and thus enable detection of developmental plasticity. Deep learning approaches such as that used here will become increasingly central to the growing field of phenomics, the acquisition of high-dimensional data on an organism-wide scale (Houle et al., 2010). Based on this study, deep learning is a powerful solution to the challenge of identifying the timing of events in developing embryos, and this has broad relevance to research across the life and medical sciences.

Dev-ResNet is a comparatively small and efficient model, consisting of just ∼5.2 million parameters and ∼3.7 GFLOPs (Giga floating point operations), respectively. It enables both fast training (320 videos s^−1^) and inference (541 videos s^−1^) times on consumer hardware (NVIDIA RTX 3090 GPU), but also achieves high performance using a moderately sized dataset. These features increase the accessibility to researchers wanting to train models for specific species, developmental periods or events, while reducing the resource cost of both training and inference (Meijering, 2020; Hallou et al., 2021; Laine et al., 2021). Generating annotated images or videos for training datasets is a major barrier in deep learning. Thus, a key goal in creating Dev-ResNet was to train an effective model using volumes of data that are attainable for those not working with model systems. Our approach to creating training data involved manual identification of developmental event timings of just 67 embryos (∼15% of total replicates imaged in the present study) to produce an accurate, generalisable model capable of accurate inference across a large dataset of 405 embryos.

A potential limitation of Dev-ResNet is its operation on one temporal stride, rather than integrating videos of different temporal strides for classification. An example of this is Google's MoViNet (Kondratyuk et al., 2021), consisting of two 3D CNNs, each processing either a ‘fast’ or ‘slow’ video input, thereby integrating the different speeds of motion present within the video. However, this model type significantly increases computational cost compared with a single 3D CNN. Another limitation of classification models generally is imbalances between classes. Though we lessened the impact of class imbalances using selective data augmentation, this could not achieve comparable accuracy to developmental events with much more original samples (Fig. 1D). Similar limitations have been observed in other studies (e.g. Liu et al., 2019). An alternative could be to train event-specific models, such as a binary classification model to identify whether a single event occurred or not. However, this approach would linearly reduce training and inference efficiency with the number of event classifications required.

Automated image and video analysis in biology will continue to advance our understanding of complex biological systems (Royer, 2023). We suggest that deep learning models such as Dev-ResNet are pivotal in broadening deep learning's application beyond traditional model species, thereby increasing its relevance to areas such as evolutionary studies of heterochrony, ecotoxicology and assessing biological sensitivity to environmental stressors. Extending deep learning approaches to more biological systems will undoubtedly drive scientific advances and, owing to its scalability, contribute to both advancing phenomics and unlocking the phenotyping bottleneck.

## Supplementary Material

10.1242/jexbio.247046_sup1 Supplementary information
