# Supplementary material for: Dev-ResNet: automated developmental event detection using deep learning
Source: J Exp Biol. 2024 May 29;227(10):jeb247046. doi: 10.1242/jeb.247046 (PMC11152166; doi:10.1242/jeb.247046)
Supplement: Supplementary information [file jexbio-227-247046-s1.pdf]

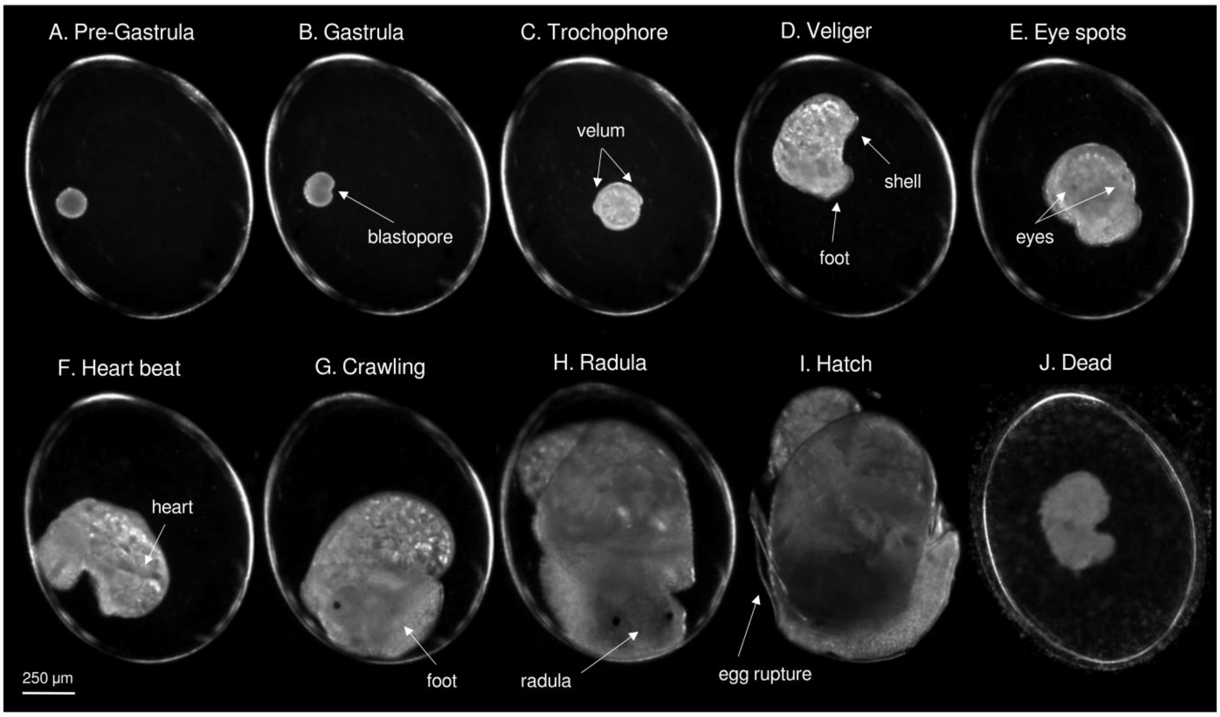


**Fig. S1.** Developmental events used in this study are shown (A-D), with key characteristics of each event indicated by the white arrows and text.

Journal of Experimental Biology • Supplementary information

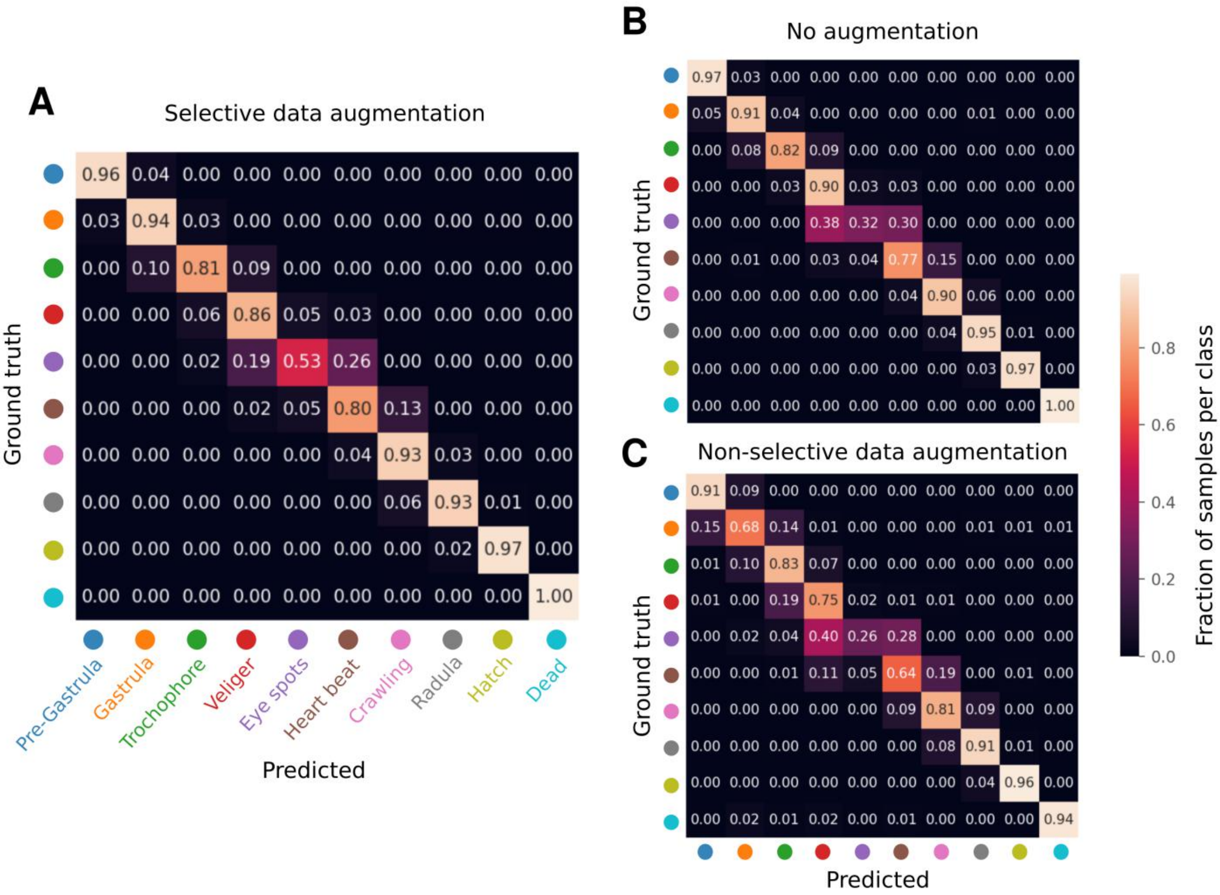


**Fig. S2.** Confusion matrices comparing counts between ground truth and predicted developmental event labels on the testing dataset for Dev-ResNet trained with no visual augmentations (B), with non-selective augmentation, i.e. overall training samples increased but class imbalances remained (C) and with selective data augmentation, i.e. class imbalances were removed by applying augmentation to varying degrees depending on a given class' representation in the original data (A). The axis tick colours assigned to each event are described in (A).

Journal of Experimental Biology • Supplementary information

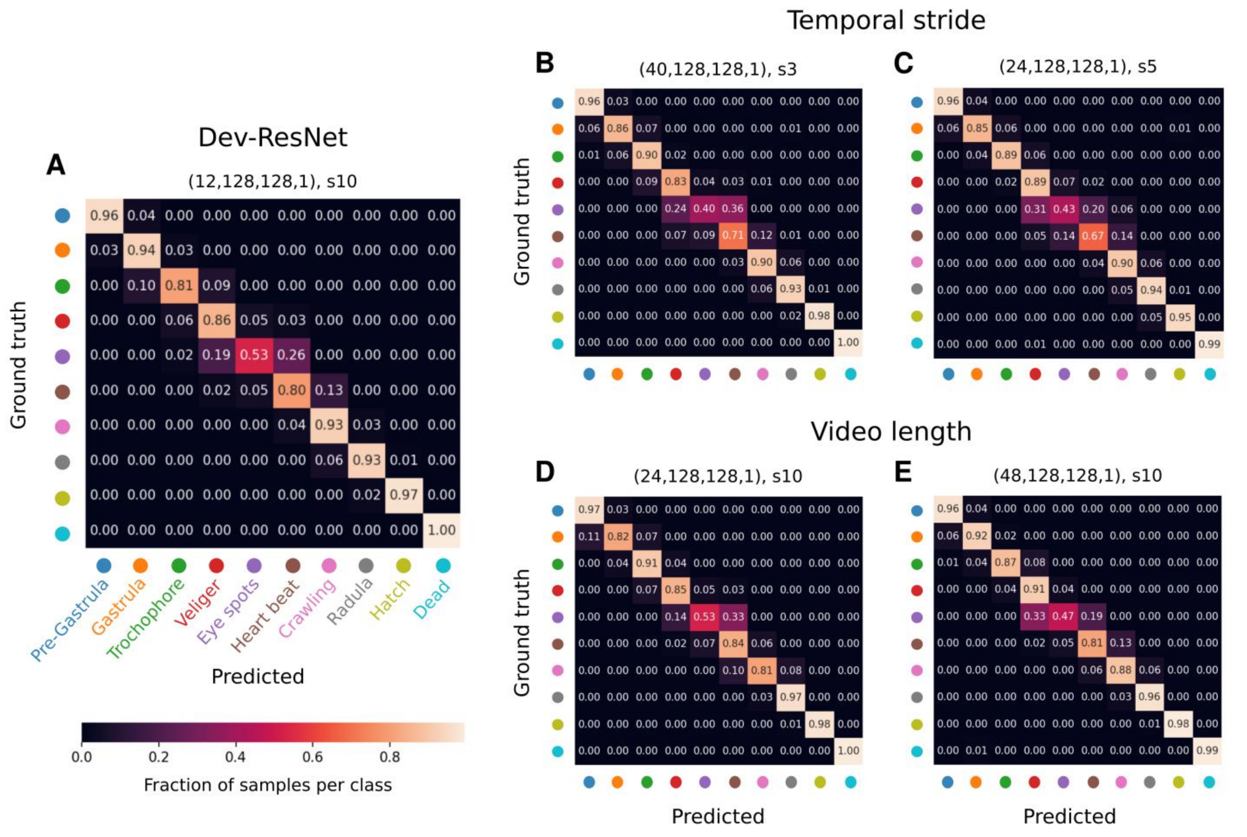


**Fig. S3.** Confusion matrices comparing counts between ground truth and predicted developmental event labels on the testing dataset for Dev-ResNet trained with different temporal strides: 3 (B), 5 (C) and 10 frames (A), but also with different video lengths: 12 (A), 24 (D) and 48 frames (E) using the same temporal stride (10 frames). The axis tick colours assigned to each event are described in (A). Input shape (length, width, height, channels) and temporal stride notation (e.g. s10 equals a stride of 10 frames) corresponding to each model are subtitles above each plot.

**Table S1.** Descriptions of developmental events examined for *Lymnaea stagnalis,* after Meshcheryakov (1990) and Smirthwaite et al., (2007), including event timings observed at a reference temperature of 20°C. Pre-Gastrula timings are excluded because we use this descriptor for the developmental period prior to gastrulation and not as an event itself, and timings of mortality ('Dead') are omitted because no mortality was observed at a reference temperature of 20°C.

| Developmental event | Description of event | Event time (hours) at 20°C (mean ± sd) |
|---|---|---|
| Pre-Gastrula | Encompasses cell divisions and flattening of embryo at poles. | N/A |
| Gastrula | Clear visible formation of blastospore and cilia driven rotation is now observable. | 49.2 ± 3.9 |
| Trochophore | Liver cells form a helmet-like velum over the embryo. | 89.0 ± 5.0 |
| Veliger | Distinct formation of the two poles of the embryo into the shell and the foot, with each pole now being clearly distuingishable. | 122.5 ± 9.5 |
| Eye spots | Pigmentation of the eye spots can be observed on the head, and they continue to darken as the embryo develops. | 143.9 ± 4.0 |
| Heart beat | A rhythmic beat can be observed in the heart situated in the shell of the embryo. The heart gradually moves toward the head of the embryo as it grows. | 150.9 ± 3.2 |
| Crawling | The foot of the embryo attaches to the egg capsule, enabling it to crawl. | 176.8 ± 10.3 |
| Radula | The radula, located in the head between the eye spots, begins to suck in capsular fluid. | 239.2 ± 3.2 |
| Hatch | Rupture of the egg capsule, followed by the emergence of the embryo. | 295.4 ± 3.7 |
| Dead | Cessation of development and movement, often accompanied by rapid growth of microbial organisms in the egg. | N/A |

## References


Meshcheryakov, V. N. (1990). The common pond snail Lymnaea stagnalis. In *Animal Species for Developmental Studies* (ed. T. A. Detlaf and S. G. Vassetzky), pp. 69-132. Consultants Bureau.

Smirthwaite, J. J., Rundle, S. D., Bininda-Emonds, O. R. and Spicer, J. I. (2007). An integrative approach identifies developmental sequence heterochronies in freshwater basommatophoran snails. *Evol. Dev.* **9**, 122-130. doi:10.1111/j.1525-142X.2007.00143.x
